# Patient perspectives on the impact of appearance and weight changes attributed to systemic glucocorticoid treatment of rheumatic diseases

**DOI:** 10.1093/rheumatology/keaf121

**Published:** 2025-03-03

**Authors:** Stephanie J Lax, Emma Dures, Susan Bridgewater, Christine A Silverthorne, Vivien Lowndes, Pam Richards, Andreia Ferreira, Michael A Shepherd, Jill Dawson, Catherine Hill, Susan Goodman, Sarah L Mackie, Mwidimi Ndosi, Fiona A Pearce, Joanna C Robson

**Affiliations:** Department of Lifespan and Population Health, School of Medicine, University of Nottingham, Nottingham, UK; School of Health and Social Wellbeing, Centre for Health and Clinical Research, University of the West of England (UWE) Bristol, Bristol, UK; Rheumatology Department, University Hospitals Bristol and Weston NHS Foundation Trust, Bristol, UK; School of Health and Social Wellbeing, Centre for Health and Clinical Research, University of the West of England (UWE) Bristol, Bristol, UK; Rheumatology Department, University Hospitals Bristol and Weston NHS Foundation Trust, Bristol, UK; School of Health and Social Wellbeing, Centre for Health and Clinical Research, University of the West of England (UWE) Bristol, Bristol, UK; Rheumatology Department, University Hospitals Bristol and Weston NHS Foundation Trust, Bristol, UK; Department of Lifespan and Population Health, School of Medicine, University of Nottingham, Nottingham, UK; School of Health and Social Wellbeing, Centre for Health and Clinical Research, University of the West of England (UWE) Bristol, Bristol, UK; Rheumatology Department, University Hospitals Bristol and Weston NHS Foundation Trust, Bristol, UK; Rheumatology Department, University Hospitals Bristol and Weston NHS Foundation Trust, Bristol, UK; School of Health and Social Wellbeing, Centre for Health and Clinical Research, University of the West of England (UWE) Bristol, Bristol, UK; Nuffield Department of Population Health, University of Oxford, Oxford, UK; Discipline of Medicine, The University of Adelaide, Adelaide, Australia; Rheumatology Unit, Royal Adelaide Hospital, Adelaide, Australia; Department of Rheumatology, The Queen Elizabeth Hospital, Adelaide, Australia; Division of Rheumatology, Hospital for Special Surgery, Weill Cornell Medicine, New York, NY, USA; Leeds Biomedical Research Centre, Leeds Teaching Hospitals NHS Trust, Leeds, UK; Leeds Institute of Rheumatic and Musculoskeletal Medicine, University of Leeds, Leeds, UK; School of Health and Social Wellbeing, Centre for Health and Clinical Research, University of the West of England (UWE) Bristol, Bristol, UK; Rheumatology Department, University Hospitals Bristol and Weston NHS Foundation Trust, Bristol, UK; Department of Lifespan and Population Health, School of Medicine, University of Nottingham, Nottingham, UK; Department of Rheumatology, Nottingham University Hospitals NHS Trust, Nottingham, UK; National Institute for Health Research, Nottingham Biomedical Research Centre, Nottingham, UK; School of Health and Social Wellbeing, Centre for Health and Clinical Research, University of the West of England (UWE) Bristol, Bristol, UK; Rheumatology Department, University Hospitals Bristol and Weston NHS Foundation Trust, Bristol, UK

**Keywords:** appearance changes, weight gain, glucocorticoids, quality of life, mental health

## Abstract

**Objective:**

To explore patients’ perspectives on the impact of appearance changes attributed to glucocorticoid treatment.

**Methods:**

A secondary inductive thematic analysis was conducted of the ‘Steroid PRO’ semi-structured interviews with patients with rheumatic conditions receiving glucocorticoids in the UK, USA and Australia.

**Results:**

Sixty patient interviews were analysed. Patient age was 26–84 years; 39 (65%) were female; and the patients had systemic vasculitis (*n* = 19), inflammatory arthritis (*n* = 14), crystal arthropathy (*n* = 2), connective tissue disorders (*n* = 16) or other/multiple (*n* = 9). In addition to participants expressing the need for more information and support, three over-arching themes were identified: (i) societal norms [‘I think my main concern, particularly being female, was the weight gain that the steroids had’ (female aged 26–30)]; these included real or perceived expectations to which participants felt pressure to conform, which were sustained through interactions with others; (ii) harms to mental health and sense of self [‘It makes you feel down. It makes you feel depressed. You don’t want to socialize because you’re not you’ (male aged 61–65)]; glucocorticoids were described as making participants ‘not look like’ themselves, associated with changes in mood and self-confidence; and (iii) burden of adjustments [‘I have a wardrobe right now that goes four different sizes’ (female aged 51–55)]; other adjustments related to diet, exercise, work, hobbies, activities of daily living and key life events.

**Conclusion:**

Patients attribute a variety of impacts on their quality of life to glucocorticoid-related appearance changes. We suggest ways to meet patients’ needs for information and support, which can be developed through further work.

Rheumatology key messages

- Societal norms shape how appearance changes due to glucocorticoid treatment impact individuals.
- These appearance changes harm individuals’ mental health and sense of self.
- Appearance changes carry a burden of adjustments for individuals to make.

## Introduction

Glucocorticoids are prescribed for a range of medical conditions, including the inflammatory rheumatic diseases. Known side effects necessitate an approach of seeking to minimize dose and duration once disease control is achieved, or use of effective and safe alternatives where possible. However, many patients with rheumatic conditions still require therapy with systemic glucocorticoids for life or organ threatening disease, or to control symptoms quickly [1–3].

Healthcare professionals are concerned about serious toxicities due to glucocorticoids across multiple systems of the body [4]. Whilst there is clinical awareness of the risk of weight gain, this is not well researched in terms of the impact on patients [5]. In a systematic review of patient perspectives on glucocorticoid use, weight gain was the concern most frequently mentioned (74% of included studies), with changes to skin, facial features, appetite and general appearance also important [6]. In a survey of people with inflammatory myopathy, 86% reported weight gain [7].

Appearance is often key to defining sense of self, and individuals’ view of themselves may be a key factor in developing depression [8]. Depression (past or current) is the most common comorbidity in rheumatoid arthritis, affecting around 15% of patients [9]—which is a two to three times greater prevalence than in the general population [10]. In patients with giant cell arteritis, glucocorticoid use and older age are predictors of anxiety and depression [11]. Depression is also associated with a higher risk of adverse health outcomes, including increased disease activity and pain perception [12, 13].

The need to incorporate patients’ perspectives on glucocorticoids in clinical practice and trials has been recommended [14, 15], resulting in the glucocorticoid-specific patient-reported outcome measure (PROM), the ‘Steroid PRO’ [16, 17]. During the development of candidate items for the Steroid PRO, the importance of appearance changes to participants was highlighted by patient interviewees and patient research partners.

The purpose of this secondary analysis is to explore more fully these issues of appearance and weight change attributed to glucocorticoids in patients with rheumatic conditions.

## Methods

We describe our methods according to the Standards for Reporting Qualitative Research [18].

### Primary analysis

To develop candidate items for the Steroid PRO, semi-structured qualitative interviews were conducted with 60 rheumatology patients in the UK, USA and Australia with the aim of exploring health-related quality of life in participants who had taken glucocorticoids. Interview design and conduct is reported elsewhere in full [16]. In brief, adult participants with a clinical diagnosis of a rheumatic condition treated with glucocorticoids within the past 2 years were recruited from rheumatology clinics. Purposive sampling ensured participants represented a broad variety of demographic characteristics, rheumatic conditions and glucocorticoid dose [16]. A conceptual framework and topic guide based on patient involvement and literature review underpinned interviews facilitated by experienced qualitative researchers without clinical experience treating patients with glucocorticoids (see Supplementary Table S1, available at *Rheumatology* online). One interviewer was a White female aged 50–60, the other a White male aged 50–65. The study complies with the Declaration of Helsinki, with ethical approval obtained in the UK (REC ref: 19/SW/0221), USA (IRB ref: 2019–0215) and Australia (CALHN ref: 12903). All participants provided written informed consent.

### Secondary analysis

In the present study, secondary analyses of all transcripts from the primary study were performed. The study team consisted of researchers, healthcare professionals and patient partners. Data were organized using NVivo version 14 [19]. Thematic analysis proceeded according to the Braun and Clarke framework [20].

S.J.L. reviewed the original findings focusing on appearance changes and undertook the secondary analysis. S.J.L. has no clinical experience treating patients with glucocorticoids, but consideration was given to the potential influence of her prior work investigating topical glucocorticoids. Processes were established such as keeping a reflexive journal and scheduling regular discussions with co-authors to ensure transparency during analysis and reporting of the findings.

First, S.J.L. conducted dataset familiarization, highlighting salient content in the transcripts using NVivo annotations. Coding then proceeded inductively, resulting in both semantic (more literal) and latent (more inferred) codes. Patterns of meaning were proposed by S.J.L. These were iterated through several rounds of discussion with J.C.R. (rheumatologist and researcher) and E.D. (psychologist and methodologist), formalized into themes and subthemes, and labelled according to meaning and analytic direction. These themes were further developed with patient research partners, A.F. and members of the RECORDER Public Partnership at a virtual meeting. Illustrative quotations were selected from the data extracts under each code to evidence each theme and subtheme, ensuring a diversity of represented participants. The study team also designed an infographic for health professionals to guide supportive conversations with patients starting glucocorticoid treatment. Whilst not a formal co-design process, feedback was sought through the RECORDER Public Partnership and by distributing postcards to both clinicians and public partners at a number of rheumatology events in 2024.

## Results

The transcripts of 60 in-depth interviews from the UK (*n* = 34), USA (*n* = 10) and Australia (*n* = 16) with patients treated with glucocorticoids for their rheumatic condition were analysed. Table 1 summarizes the demographic and disease characteristics [2]. Two-thirds of participants reported weight gain, one-third skin thinning, with a range of other skin, hair and nail changes also reported.

**Table 1.** Summary of demographic data for interviews with 60 patients treated with glucocorticoids for their rheumatic condition

Characteristic	*n*
Country	
UK	34
Australia	16
USA	10
Age	
18–39 years	14
40–59 years	15
60–79 years	26
80+ years	5
Sex	
Female	39
Male	21
Ethnicity	
Asian	2
Black	4
Mixed/multiple ethnic groups	2
White	51
Other	1
Condition	
Systemic vasculitis	19
Inflammatory arthritis	16
Crystal arthropathy	2
Connective tissue disorders	17
Other	10

Details of the specific conditions studied can be found in Supplementary Table S2, available at *Rheumatology* online.

**Figure 1.**
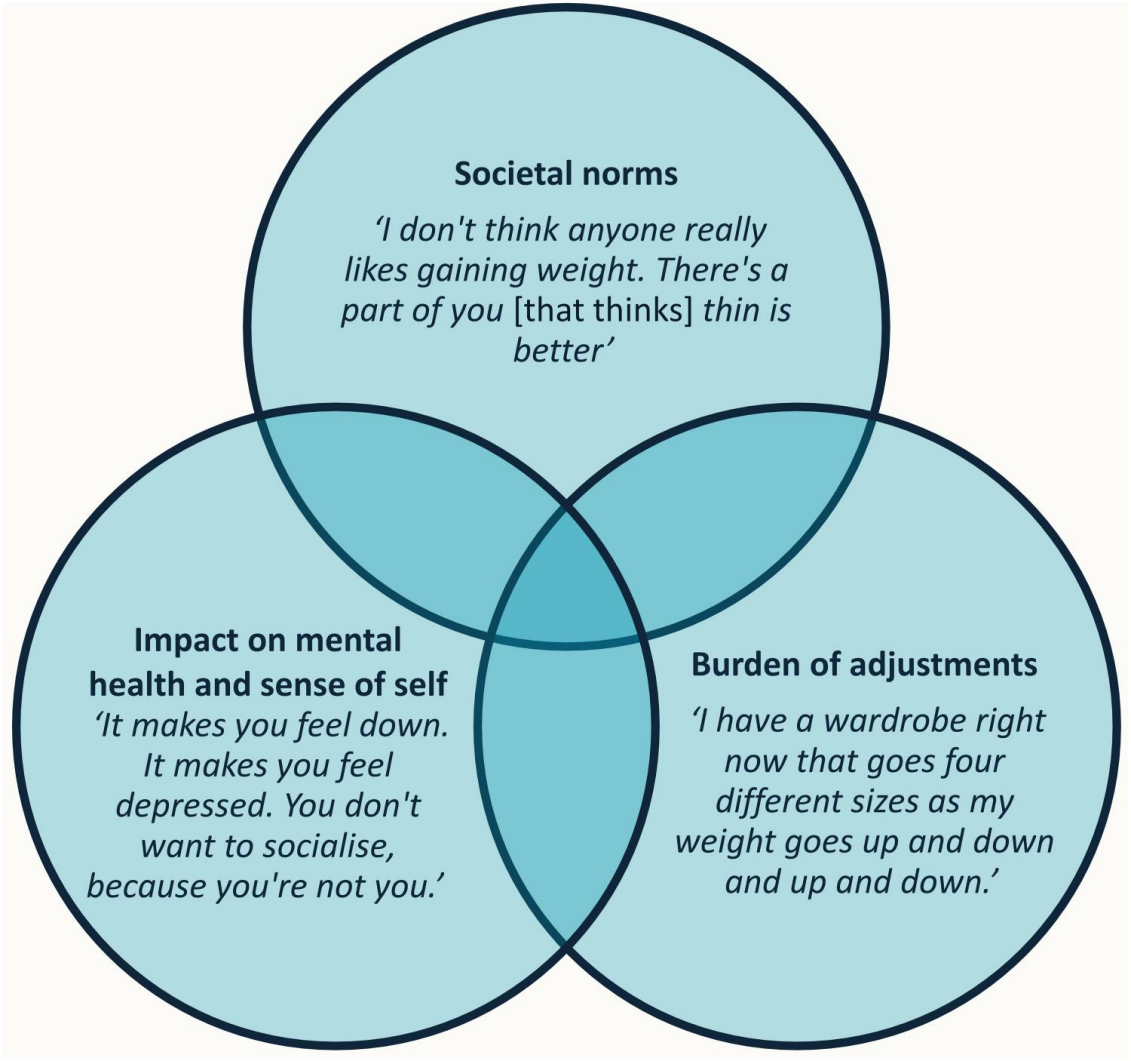
Impact of appearance changes due to glucocorticoid treatment in the rheumatic diseases

The analysis was organized into three overarching themes to describe the impact of appearance and weight change on participants (see Fig. 1): (i) societal norms shape how appearance changes impact individuals, (ii) appearance changes harm individuals’ mental health and sense of self, and (ii) appearance changes carry a burden of adjustments for individuals to make.

### Theme 1: societal norms shape how appearance changes impact individuals

**Figure 2.**
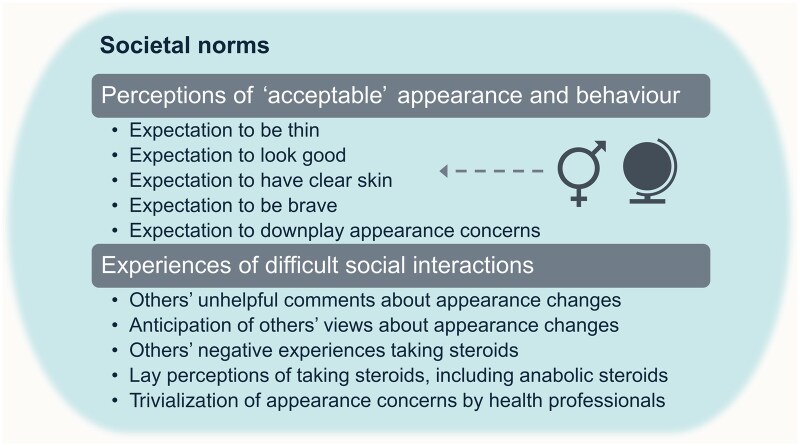
Impact of societal norms on appearance change (theme 1). Gender and globe symbols indicate that expectations may vary according to gender and cultural background, which are areas for testing in future research

An individual’s notion of whether they ‘look good’ may be calibrated by societal norms comprising real or perceived expectations and sustained through interactions with others. The mental picture people believe others have of them may be as important as their mental picture of themselves [21]. Related subthemes, all suggesting negative impacts, are summarized in Fig. 2.

#### Perceptions of acceptable appearance and behaviour

Participants conveyed expectations they felt concerning what constituted ‘an acceptable appearance’ reflecting dominant cultural narratives: ‘I don’t think anyone really likes gaining weight. There’s a part of you [that thinks] thin is better’ [AD-GC14, female, mixed/multiple ethnicities, aged 26–30, granulomatosis with polyangiitis (GPA)]. As taking glucocorticoids is associated with weight gain, individuals find it more difficult to aspire to this ideal of thinness, and may feel negatively judged by themselves and society.

It was suggested that expectations may vary depending on cultural background and gender. Specific examples concerned hair loss: ‘Being an Italian, I had lots of hair for all my life. When I started losing it, it was a bit of a worry’ (AD-GC03, White female aged 66–70, inflammatory myositis). Another example concerned weight: ‘I think my main concern, particularly being female, was the weight gain’ (AD-GC14, female, mixed/multiple ethnicities, aged 26–30, GPA).

Expectations on acceptable behaviours were also reported, including downplaying appearance concerns: ‘I mean that’s a purely vanity thing, I know’ (NY-ID09, Black female aged 26–30, inflammatory myositis). This indicates participants felt appearance concerns are not perceived as legitimate causes for distress. A need to be brave was also expressed ‘I needed to keep working to try and not make myself feel like I was a complete victim’ (AD-GC03, White female aged 66–70, inflammatory myositis).

When individuals consider themselves failing to meet expectations, this may incite feelings of shame. Furthermore, as individuals both shape and are shaped by societal norms, these expectations may be perpetuated.

#### Experiences of difficult social interactions

Many participants reported receiving unhelpful comments: ‘She said “You look ever so well, ever so *well*”, and I just said to her, I said “No”, I said “You mean I look fat”, and she said, “Well I didn’t like to say anything”, but she did’ (BR-06-U0010, White female aged 76–80, giant cell arteritis (GCA)). Others acknowledged their own judgements on others’ negative experiences with glucocorticoids and anticipation of others’ views: ‘She had an old skin before her time … Yeah, I don’t want to look like that’ (BR-10-U0016, White female aged 61–65, RA), and ‘So I have noticed acne on my chin area … I can notice it and I’m sure other people could notice it, too’ (NY-ID09, Black female aged 26–30, inflammatory myositis).

Participants described hearing unwelcome opinions on whether to take steroids, sometimes assuming anabolic steroids rather than glucocorticoids: ‘When you take steroids people immediately say oh my God, you are going to become Arnold Schwarzenegger’ [AD-GC06, Asian female aged 26–30, systemic lupus erythematosus (SLE)]. Some participants received support from family and friends: ‘I was really lucky I’m married, and my husband is, has been just fantastic … And lying to, to me about it, when I’m like look how big my face is, he’s like no, it’s not, you look amazing’ (BR-12-U0019, White female aged 31–35, Behçet’s disease), and ‘They just try to get me out of the house. They try to get me to socialize’ (NY-ID02, Hispanic female aged 26–30, SLE). However, some reported negative interactions with health professionals when they felt their appearance concerns were trivialized: ‘I said “I look in the mirror and I see it’s not me” … and he said, “Oh you look the same as I’ve seen you before”, and I thought, don’t lie, I don’t’ (BR-06-U0010, White female aged 76–80, GCA).

### Theme 2: appearance changes harm individuals’ mental health and sense of self

When an individual’s appearance is compromised, their mental health and sense of self may be also. Participants described a strong link between appearance and feelings: ‘It does really affect, you know, how you feel and kind of your state of mind and things’ (BR-16-U0028, White female aged 36–40, Takayasu arteritis), and ‘When I got off them … the moon face disappeared, and then the fluid retention dropped off, and I fit back into my normal clothes. I just felt a lot better. I felt free’ (AD-GC16, White male aged 61–65, inflammatory myositis). Subthemes are described below.

#### Loss of identity

Appearance changes reportedly made participants feel less like themselves: ‘I haven’t got a very big face, and it was really puffy looking, and it wasn’t me, sort of thing’ (AD-GC04, White female aged 66–70, inflammatory myositis), and ‘It also affected the way I felt, my image, how I looked’ (NY-ID06, Hispanic male aged 31–35, SLE). Some participants also reported feeling less able to express themselves: ‘I love wearing dresses, and in the summertime, I won’t do it unless they’re long dresses because of the bruising and the scarring on my legs’ (NY-ID10, White female aged 51–55, SLE).

#### Low self-confidence

Several participants reported decreased self-confidence due to appearance changes: ‘But inside, prednisolone and the effects on my body have made me very, very insecure’ (AD-GC06, Asian female aged 26–30, SLE), and ‘The moon face makes you become insecure because you have a face of a tomato’ (AD-GC01, White female aged 66–70, GCA).

#### Detrimental mood changes

Some participants reported heightened emotions: ‘Larger doses, weight gain, the whole package. It just makes you more emotional’ (NY-ID10, White female aged 51–55, SLE), and ‘I was quite short tempered … I just wanted to hide away from people, because my appearance changed pretty much overnight’ (BR-30-U0051, male, mixed/multiple ethnicities, aged 51–55, sarcoidosis). Conversely, some participants reported feelings associated with low mood that specifically related to appearance changes, such as hair loss and body shape changes: *‘*My hair is thinning dreadfully … My hair is—yeah, it’s very disappointing’ (AD-GC01, White female aged 66–70, GCA), and ‘My face has got that puffy moon look about it and it alters the shape of your body … it is a bit depressing’ (BR-25-U0043, White male aged 76–80, GPA). Such mood changes may be linked to feelings of shame, likely compounded by the burden of their chronic condition.

Some participants indicated more support, including from peers, and information may have been helpful to prepare them for the effects of glucocorticoids: ‘So you become a little depressed because you just don’t know … they give the paperwork and everything, but reading something and actually talking to someone that actually has been through it is a [totally] different experience’ (NY-ID09, Black female aged 26–30, inflammatory myositis).

#### Triggering of pre-existing eating disorders

Two participants reported their weight changes triggered pre-existing eating disorders: ‘I was starving myself even more while on prednisolone, which is why I was skinny’ (AD-GC06, Asian female aged 26–30, SLE), and ‘One of the other problems is in the past, I’ve had um issues with Bulimia, um, which then mentally played on me’ [BR-20-U0036, White female aged 36–40, eosinophilic granulomatosis with polyangiitis (EGPA)].

#### Worries about recurrent appearance changes

Participants reported that concerns about appearance changes affected their attitude to taking steroids in future: ‘I think my mental health probably did suffer a bit when I was on the higher doses, then when I went down and went back up again, just because I knew what was coming with the side effects’ (AD-GC14, female, mixed/multiple ethnicity, aged 26–30, GPA), and ‘You do not want to go through those symptoms again, the moon face. I’m slowly coming down from that looking puffy, like a little marshmallow walking down the road … The acne, no one wants to go through that. The weight gain, the change of your emotions and your attitude, no one wants to go through that’ (NY-ID09, Black female aged 26–30, inflammatory myositis).

### Theme 3: appearance changes carry a burden of adjustments for individuals to make

When an individual experiences a change in appearance, they may actively seek to restore what is normal for them. There may be a considerable burden of adjustments for patients in relation to appearance changes, in addition to the burden of living with a chronic rheumatic condition. A list of adjustments reported by participants are given in Table 2, with two key adjustments explored in more detail below:

**Table 2.** Appearance change and range of adjustments required (Theme 3)

Appearance changes carry a burden of adjustments for individuals to make
Increasing physical activity
‘So obviously I'd have to exercise more and things like that, and eat better and things like that, to get that weight off’ [AD-GC14, female (mixed/multiple ethnicity) aged 26–30 with GPA]
Improving diet
‘I’m not massively slim, but I try really hard, that’s just become a way of life now … I’m very careful about what I eat’ [BR-30-U0051, male (mixed/multiple ethnicity) aged 51–55 with sarcoidosis]
‘I mean on the weight thing I do struggle with my weight and I do that fasting diet, erm 5:2’ (BR-08-U0013, White female aged 71–75 with GCA)
Taking care to avoid knocks
‘I do work in the garden and I now and again knock myself and I bleed very easily, as I've said. So I'm being more careful’ (AD-GC11, White male aged 31–35 with SAPHO syndrome)
Reduced working or working from home
‘The fact that I lost my hair was another big impact on how I felt about going out and being anywhere. That impacted on me greatly. I took a bit of time off work, but I could work from home as well’ (AD-GC03, White female aged 66–70 with inflammatory myositis)
Reduction in hobbies
‘We used to do a lot of socializing, dancing, etc., which I’ve found now I’m reluctant to do’ (BR-25-U0043, White male aged 76–80 with GPA)
Postponing important events
‘I was supposed to graduate in May but I might put that back until I … so I’ll look okay, hopefully okay on the photos ’cause I don’t really want to be, you know …’ (BR-16-U0028, White female aged 36–40 with Takayasu arteritis)
Purchasing additional clothing
‘So, it just is very frustrating because then you have to buy more clothes. I have a wardrobe right now that goes four different sizes as my weight goes up and down and up and down’ (NY-ID10, White female aged 51–55 with SLE)
Applying creams and oils
‘I’m starting to get stretch marks now … So I’m trying to use alternatives to prevent stretch marks … little things to try to moisturize my skin …’ [NY-ID09, Black female aged 26–30 with inflammatory myositis (polymyositis or dermatomyositis)]
‘Yes, I’ve got very dry skin; my skin has dried out like snakeskin almost; I mean I use pots and pots and pots of moisturizer’ (BR-08-U0013, White female aged 71–75 with GCA)

GCA: giant cell arteritis; GPA: granulomatosis with polyangiitis; RA: rheumatoid arthritis; SAPHO: synovitis, acne, pustulosis, hyperostosis, osteitis; SLE: systemic lupus erythematosus.

### Adjustments around physical activity

Several participants reported they needed to exercise more to mitigate weight gain: ‘Walks, being active is a very important thing, being on steroids. Even if you can’t be a bodybuilder, it’s okay, but like little walks, ten minutes’ (NY-ID09, Black female aged 26–30, inflammatory myositis). Improved mobility associated with better disease control facilitated taking exercise for some: ‘Because I was not in pain, I was getting a little bit more erm, I was walking and getting a bit more active’ (BR-13-U0020, White female aged 71–75, polymyalgia rheumatica).

However, some participants found weight gain impacted their mobility and therefore their ability to exercise: ‘It’s staved my mobility because I was heavier again’ (BR-27-U0045, White female aged 71–75, RA), and ‘With the high dose of prednisone since I gained weight, I get really tired if I try to do like exercise or something. And I used to be very sporty, so that just completely changed’ (NY-ID02, Hispanic female aged 26–30, SLE). This reportedly contributed to a vicious cycle with further weight gain: ‘I think that the weight gain was a gradual thing … But as it got worse, exercise become less and very hard … and the more cortisone I took to control my condition, to be able to do something, meant that I gained more weight. The consequences of that [are] less exercise, don’t feel well, high blood pressure’ (AD-GC10, White male aged 71–75, palindromic rheumatoid). The burden of having a chronic condition associated with fatigue and pain also limited the ability to exercise: ‘Because of the lupus, it’s very hard to exercise’ (BR-32-U0053, White male aged 36–40, SLE).

### Adjustments to diet

Many participants reported making changes to their diet, including subscribing to weight loss initiatives: ‘But I did read up to be careful with your salt intake, so I do remember cutting out a lot of salt, because it makes you retain fluid’ (AD-GC16, White male aged 61–65, inflammatory myositis), and ‘I went to Weight Watchers while I was on the steroids, because I wanted to lose weight’ (AD-GC04, White female aged 66–70, inflammatory myositis).

Some participants valued information received from health professionals to mitigate weight gain through diet: ‘The rheumatologist I saw first, he said it’s not the actual steroids that put the weight on, it’s just it gives you the appetite to eat more. It’s still really up to you whether you eat more’ (AD-GC04, White female aged 66–70, inflammatory myositis). One participant obtained helpful information from fellow gym users: ‘I think the thing that helped the most was probably the gym. Because when you go to the gym you talk to people who know nutrition’ (AD-GC06, Asian female aged 26–30, SLE). It was suggested that offering information should be done tactfully: ‘I would get really angry if someone came up to me and was like you know you shouldn’t eat so much because you are on steroids’ (AD-GC06, Asian female aged 26–30, SLE).

As for physical activity, the burden of the underlying condition was reported to limit participants’ ability to improve their diet: ‘Even though I tried not to have biscuits in the house … because I was in pain I comfort ate and that didn’t help’ (BR-27-U0045, White female aged 71–75, RA and polymyalgia rheumatica).

### Infographic for healthcare professionals

**Figure 3.**
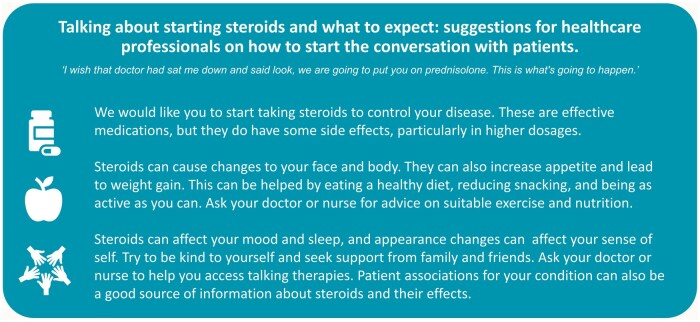
Infographic to support healthcare professionals in talking to patients about the impact of glucocorticoids or ‘steroids’

Involvement of patient partners and a nurse specialist was integral in interpreting themes important to patients. An infographic to support health professionals when talking to patients (Fig. 3) was also developed based on needs identified in the participant interviews and patient partner feedback: ‘I wish that doctor had sat me down and said look, we are going to put you on prednisolone. This is what’s going to happen. You are going to get very, very hungry’ (AD-GC06, Asian female aged 26–30, SLE), and ‘More support needs to be ongoing in terms of supervision. Now, I am aware of how busy doctors are but surely there must be some practice nurses and people like that who could do this role within a GP practice’ (BR-07-U0011, White female aged 71–75, RA).

The infographic was designed for health professionals to support them to start conversations with patients starting glucocorticoid treatment, including how to address issues of potential weight and appearance change in a sensitive way. It has been received favourably by public partners.

## Discussion

This secondary analysis of interview data from patients with a range of rheumatic conditions [16] focused in depth on the impacts of appearance and weight changes attributed to glucocorticoids. Three overarching themes were identified: the impact of society on people experiencing appearance and weight changes due to glucocorticoids, how this can impact peoples’ confidence and sense of self, and the list of additional adjustments that people may need to make to cope with appearance and weight change.

Changes in appearance can have a direct impact on mental health and sense of self. The link between depression and appearance-related social anxiety has been shown previously in rheumatoid conditions, with poor social support also being associated with greater depressive symptoms [22, 23]. In turn, depression may lead to avoidance of social interactions, further reducing social support [22]. It has been suggested that depression itself may also increase appearance concerns [23]. Weight gain has also been associated with vulnerability to disordered eating behaviours across several chronic conditions [24–28], and this was a concern raised by participants in this study. Researchers exploring patient perspectives of glucocorticoids in ANCA-associated vasculitis have previously reported on appearance changes and strategies to mitigate against them [29]. In this analysis and the primary Steroid PRO analysis it was seen that changes in weight could impact interpersonal relationships and lead to unwanted enquiries about peoples’ health, as their disease was suddenly more ‘visible’ [16].

This study identified a substantial list of adjustments participants made to mitigate or adapt to their appearance change. These may be considerable in addition to the burden of living with a chronic rheumatic condition. Although rarely explicitly mentioned by participants, many of these adjustments carry cost implications (e.g. gym or diet plan membership, purchasing additional clothing), which may affect individuals differently depending on their financial circumstances.

In the UK, The National Institute for Health and Care Excellence recommends that body weight and body mass index should be measured before commencing long-term treatment with oral glucocorticoids and monitored [30]. This could be an opportunity to raise the potential of weight and appearance change. Feedback from our patient research partners and interview participants highlighted that this topic was rarely discussed by doctors or nurses, meaning people were shocked and felt unprepared. An infographic was therefore designed to help health care professionals start these conversations. Advice should be tailored depending on how ill an individual is and their circumstances, especially for whom this may be a sensitive area. For example, some patients may lack an established support network (e.g. have recently moved to the area or live alone), lack the means to access paid services (e.g. diet plans or gym membership), or have a history of mental health or eating disorders that should be considered. Individuals may also benefit from hearing from people with similar lived experiences, for example through the Health Experiences Insights website [31].

There was broad consensus when triangulating the interpretations of data extracts between clinical co-authors and patient research partners in this study, but there were some interesting differences. Clinicians believed glucocorticoids usually increased body hair, whilst interviewees and patient partners felt strongly that hair loss was a feature, unexplained by active disease or medications such as methotrexate. This example highlights that both patient and clinician perspectives are important to consider, particularly in supporting shared care when making decisions about steroid reduction or use of alternative medications based on priorities important to patients [32, 33].

The main strength of this study is the large international qualitative dataset, including a range of demographic characteristics and rheumatic conditions. For example, patients were purposively sampled to ensure inclusion of male and female perspectives, which may not always be the case in rheumatology research due to the epidemiology of the individual diseases [34]. However, as a secondary analysis, our enquiry was limited by the scope of the original interviews. The work was conducted in English-speaking, high-income countries, which may reduce the generalizability of our findings. Nevertheless, our conclusions may potentially support healthcare professionals to explore and anticipate patients’ concerns and empower patients to seek information and support when they need it. Future co-design to create new materials for glucocorticoids may also be helpful.

In summary, this work has highlighted that rheumatology patients treated with systemic glucocorticoids experience a variety of impacts on their mental health and sense of self because of appearance changes. Further work is needed to better understand the mechanisms leading to these appearance changes and to improve the provision of information and support available to patients taking glucocorticoids for their rheumatic condition.

## Supplementary Material

keaf121_Supplementary_Data

## Data Availability

The data underlying this article will be shared on reasonable request to the corresponding author.
