# Supplementary material for: Patient perspectives on the impact of appearance and weight changes attributed to systemic glucocorticoid treatment of rheumatic diseases
Source: Rheumatology (Oxford). 2025 Mar 3;64(6):3854–62. doi: 10.1093/rheumatology/keaf121 (PMC12107043; doi:10.1093/rheumatology/keaf121)
Supplement: keaf121_Supplementary_Data [file keaf121_supplementary_data.zip › keaf121_Supplementary_Data/rhe-24-2737-File005.docx]

**Supplementary Table S1: PRO development for glucocorticoids: interview prompts and cues (October 2019)**

The interview should be started by asking the participant very broad questions about their use and experience of glucocorticoids. The interviewer will then aim to expand on each area of interest with in-depth probing of each topic as it emerges. These prompts and cues can be referred to throughout the interview, to ensure that the full range of impacts of GCs are covered during the interview.

Introduction: Thank you for participating in this interview. You have been selected to participate in this study to explore your experiences regarding the use of steroids, which you either have used in the past and/or are currently using, and to assess the impacts of steroids on various aspects of your life. I am just going to ask you some questions about steroids (also called glucocorticoids, which can be used interchangeably for this interview) and how you have experienced taking them for your rheumatic disease.

Section A. Experience of taking steroids

*Can you tell me about your first experience of taking steroids?*

- When was this?
- Why were you given steroids?
  - Was it for the start of your disease or for a flare up?
- What dose were you on?
- Was it given by a pill or through the vein or both?
- Did the dose change increase initially or was it able to be decreased?
  - How did you feel about this change?
- What was your opinion about steroids prior to starting them?
  - How did your opinions change after you started taking them?
- How were you feeling mentally just before starting steroids?
  - How were you feeling mentally in the first few weeks of steroids?
  - How about your mental feelings after a few months? How about your mental feelings after a few years?
- How were you feeling physically just before starting steroids?
  - How were you feeling physically just before starting steroids?
  - How about your physical feelings after a few months?
  - How about your physical feelings after a few years?

*Can you tell me about other occasions when you have started or really increased your steroids?*

- When was this?
- For what reason?
- What dose were you on?
- Was it given by a pill or through the vein or both?
- Did the dose change increase initially or was it able to be decreased?
- What was your opinion of steroids in this/these occasions?
  - How was it different than prior to your first experience?
- How were you feeling mentally just before starting steroids?
  - How were you feeling mentally in the first few weeks of steroids?
  - How about your mental feelings after a few months? How about your mental feelings after a few years?
- How were you feeling physically just before starting steroids?
  - How were you feeling physically just before starting steroids?
  - How about your physical feelings after a few months?
  - How about your physical feelings after a few years?

*Have you ever been on long-term steroids (e.g. over a year)?*

- If yes, can you tell me your experience of taking steroids over a longer period?
- When was this?
- What dose was it?
- Was it given by a pill or through the vein or both?
- Did the dose change increase initially or was it able to be decreased?
- What is your opinion on taking steroids over a long time before you started them?
  - How has your opinions changed since you started taking steroids?
- How did you feel physically in the months or years after taking the steroids? (depending on how long you have been on them)
- How did you feel mentally in the months or years after taking the steroids? (depending on how long you have been on them)

*Have you ever had an intramuscular injection of steroids?*

- If yes, what was your experience?
- When was this?
- What is your opinion on getting an intramuscular shot of steroids before you started them?
  - How has your opinions changed since you received the shot of steroid?
- How did you feel physically in the months or years after getting the shot?
- How did you feel mentally in the months or years after getting the shot? (depending on how long you have been on them)

*Have you ever had a joint injection with steroids?*

- If yes, what was your experience?
- When was this?
- Which joint(s) were involved?
  - Were there multiple at once?
- What is your opinion on steroid joint injection before you started them?
  - How has your opinions changed since you received it?
- How did you feel physically in the months or years after getting the steroid injection?
- How did you feel mentally in the months or years after getting the steroid injection?

Section B. Impact

Interviewer: Now, we are going to switch gears and assess the impacts of corticosteroids on other areas of your life.

*During any of the occasions that you have taken the steroids, what physical symptoms did you experience? Please tell us any symptoms you can think of, either large or small we are interested to hear about them: Please repeat for each new physical symptom*

- When did the symptom happen (ie what dose did it occur at)?
  - How did the symptom change over time with dose changes?
- How would you describe the severity of the symptom?
  - What words would you use to describe it?
- Did the symptom have an impact on what you could do physically?
- Did the symptom have an impact on your work?
- Did the symptom have an impact on your family life or friendships?
- Did this symptom impact on personal relationships?
- Did this symptom have any impact on hobbies?
- Did this symptom impact on what tasks you could do at home?
- Did this symptom have any impact on social life?
- Did having this symptom change how you feel overall?

*During any of the occasions that you have taken the steroids, what psychological symptoms did you experience? Please tell us any symptoms you can think of, either large or small we are interested to hear about them: Please repeat for each new symptom*

- When did the symptom happen (ie what dose did it occur at)?
  - How did the symptom change over time with dose changes?
- How would you describe the severity of the symptom?
  - What words would you use to describe it?
- Did the symptom have an impact on what you could do physically?
- Did the symptom have an impact on your work?
- Did the symptom have an impact on your family life or friendships?
- Did this symptom impact on personal relationships?
- Did this symptom have any impact on hobbies?
- Did this symptoms impact on what tasks you could do at home?
- Did this symptom have any impact on social life?
- Did having this symptom change how you feel overall?

Section C: Thoughts about steroids

*What thoughts do you have about taking steroids?*

*Where do you think your thoughts have come from?*

*What do you think about increasing steroids and why?*

*What do you think about decreasing steroids and why?*

*What do you think about the future whilst you are on steroids?*

*What do you think about the future when you stop steroids?*

Section D: Help and support around use of steroids

*Would you have liked any support or education on how to deal with steroids?*

*Did anything help with the effects of steroids? (either anything you did yourself, or advice you had from others?)*

*What advice would you give someone else starting steroids?*

*Many thanks for taking part in this interview, are there any other important topics you would like to discuss about steroids?*
