# Supplementary material for: Patient perspectives on the impact of appearance and weight changes attributed to systemic glucocorticoid treatment of rheumatic diseases
Source: Rheumatology (Oxford). 2025 Mar 3;64(6):3854–62. doi: 10.1093/rheumatology/keaf121 (PMC12107043; doi:10.1093/rheumatology/keaf121)
Supplement: keaf121_Supplementary_Data [file keaf121_supplementary_data.zip › keaf121_Supplementary_Data/rhe-24-2737-File006.docx]

**Supplementary Table S2: Detailed diagnoses for 60 rheumatology patients interviewed.**

| **Condition** | **Number of Participants** |
| --- | --- |
| **Connective tissue disorder** | **16** |
| Inflammatory myositis | 7 |
| Systemic lupus erythematosus | 9 |
| **Crystal arthropathy** | **2** |
| Gout | 2 |
| **Inflammatory arthritis** | **14** |
| Ankylosing spondylitis | 1 |
| Palindromic arthritis | 1 |
| Polymyalgia rheumatica | 3 |
| Rheumatoid arthritis | 9 |
| **Other/multiple** | **9** |
| Ankylosing spondylitis and psoriatic arthritis | 1 |
| IgG4 | 1 |
| Inflammatory myositis and granulomatosis with polyangiitis | 1 |
| Rheumatoid arthritis and polymyalgia rheumatica | 3 |
| SAPHO (synovitis acne pustulosis hyperostosis osteitis) syndrome / CRMO (chronic recurrent multifocal osteomyelitis) | 1 |
| Sarcoidosis | 1 |
| Sclerosing mesenteritis | 1 |
| **Systemic vasculitis** | **19** |
| Behcet’s disease | 1 |
| Eosinophilic granulomatosis with polyangiitis | 4 |
| Giant cell arteritis | 5 |
| Granulomatosis with polyangiitis | 8 |
| Takayasu arteritis | 1 |
| **Total** | **60** |
